# Masks, Gloves, and the COVID-19 Pandemic: Rapid Assessment of Public Behaviors in the United States

**DOI:** 10.3390/epidemiologia1010004

**Published:** 2020-11-05

**Authors:** Jagdish Khubchandani, Diana Saiki, Jayanthi Kandiah

**Affiliations:** 1 Department of Public Health Sciences, College of Health and Social Services, New Mexico State University, Las Cruces, NM 88003, USA; 2 Department of Marketing, College of Business, Ball State University, Muncie, IN 47306, USA; desaiki@bsu.edu; 3 Department of Nutrition and Health Science, College of Health, Ball State University, Muncie, IN 47306, USA; jkandiah@bsu.edu

**Keywords:** coronavirus, mask, glove, prevention, COVID-19, behavior, public health

## Abstract

The COVID-19 outbreak was declared a national emergency in the U.S. in March 2020, and in April 2020, the U.S. government authorities issued recommendations on the use of masks and gloves as protective measures. Despite such recommendations, popular media reports highlighted a lack of compliance. However, no systematic study has examined the use of protective strategies (e.g., wearing a mask) by the American public to prevent the spread of COVID-19 during early stages of the pandemic. The purpose of this study was to conduct a rapid national assessment of public behaviors to prevent COVID-19 spread during the early stages of the pandemic and to assess how these behaviors may have differed based on selected sociodemographic characteristics. A total of 835 adult Americans nationwide took a multi-item survey and were asked about wearing masks, gloves, and their demographic background. The majority of the study participants reported wearing a mask more often during the pandemic (76%), but the majority did not wear gloves more often during the pandemic (30%). Significant differences (*p* < 0.05) for wearing masks were found based on sex, age, ethnicity, marital status, living arrangements, and employment status. For gloves, significant differences were found based on sex, age, marital status, and employment. While the pandemic continues to unfold and with recent reports of a surge in cases in the U.S., public health practitioners and policymakers must emphasize COVID-19 prevention strategies for the general public and explore pragmatic options to increase compliance of protective behaviors among the general public.

## 1. Introduction

The first case of COVID-19 was confirmed in the U.S. on 21 January and within three months, more than a million people tested positive and more than 100,000 died of coronavirus. With increasing morbidity and mortality and to prevent transmission of COVID-19, on 3 April 2020, the CDC encouraged the wearing of face masks by the general public and only recommended wearing gloves to frontline workers [1,2]. Despite the risk of COVID-19 infection, popular media reported that many people were apprehensive with regards to wearing or not wearing a mask or gloves. Some individuals termed wearing a mask as “culture war” and almost equivalent to a “political act”. Conflicts have escalated at essential businesses such as health care facilities and grocery stores when patients or customers refuse to enter without a face mask [3,4,5,6]. Choosing not to wear a mask is viewed by many Americans as an individual’s “right”, while others believe wearing a mask is a courtesy. A May 2020 poll conducted by the *Huffington Post*, found that more democrats (76%) compared to Republicans (51%) believed wearing a face mask protected public health, while fewer Democrats (20%) and more Republicans (42%) believed it was a matter of personal choice. Overall, Republicans had much more opposing and negative views for wearing face masks, given a variety of questions on wearing masks [5]. Concerns about being judged, racially profiled, and discriminated against were also highlighted in mass media concerning Americans wearing face masks in public places [4,6].

Despite the polls and the political discussions, no study to date has systematically examined how Americans’ face mask and glove-wearing behaviors changed during the early stages of the pandemic. Moreover, public polls did not consider aspects such as sociodemographic differences in behaviors and often rely on single-item measures that may lack reliability or validity and may not be generalizable to the entire U.S. population. Thus, the purpose of this study was to assess the prevalence of wearing masks and gloves in the general American population during the early stages of the COVID-19 pandemic and how these behaviors varied by sociodemographic characteristics.

## 2. Materials and Methods

A multi-item online survey was used which included questions to assess demographic characteristics of participants (age, sex, race, ethnicity, the region of residence, and employment status) and mask and glove-wearing behaviors. The masks and gloves section had three options for the participants to respond. These options were: “more during the pandemic—MT”, “same as before —SAB the pandemic”, and “less than before—LB the pandemic”. After approval of the study protocol by the University’s Institutional Review Board, the survey was made available on Amazon Mechanical Turk in the last week of April 2020. Before the completion of the survey, participants were informed about the objective of the study and emphasized that their participation was voluntary and anonymous.

Data were analyzed using SPSS 25. We computed descriptive statistics to describe demographic characteristics of study participants and their mask and glove-wearing behaviors (i.e., frequencies and percentages). Next, mask and glove-wearing behaviors were stratified by sociodemographic characteristics to assess group differences by using chi-square tests. Statistical significance was established at *p* < 0.05.

## 3. Results

A total of 835 adults participated in the study (Table 1), and the majority were: female (52%), white (63%), non-Hispanic (78%), working full time (55%), older than 35 years (66%), working from home (63%), and were currently living with the family (71%). Most were either married (45%) or single (40%). Nationwide geographical region of residence of the participants was as follows: South (36%); West (28%); Northeast (18%); and Midwest (17%).

With regard to wearing of masks and gloves (Table 2), the majority reported wearing a mask more often during the pandemic (76%), but the majority did not wear gloves more often during the pandemic (30%). Comparison of demographic characteristics found statistically significant differences for wearing masks with the following groups reporting a higher prevalence of mask-wearing compared to their counterparts: females (82%), divorced/widowed (85%), living with family members (81%), unemployed (85%), non-Hispanics (79%), and those ≥36 years of age (81%). Similarly, for gloves, the following groups were significantly more likely to report wearing a mask more often during the pandemic compared to their counterparts: females (59%), divorced/widowed (54%), unemployed (38%), and those ≥36 years of age (35%). While there was no statistically significant difference based on region, individuals from the Northeastern U.S. had the highest prevalence of wearing a mask (81%) or gloves (36%) more often during the pandemic.

## 4. Discussion

Irrefutable evidence exists on using masks when out in the public to prevent the spread of droplets from a person infected with COVID-19 (e.g., coughing, talking, or sneezing). With regard to gloves, it has been encouraged only for people employed in the field of healthcare or for people taking care of the sick, and for the general public, there is no conclusive evidence on benefits [7,8]. Results from this first and largest study in the U.S. shows key differences in wearing masks or gloves by the general public based on sociodemographic characteristics during early stages of the pandemic.

Despite the evidence and recommendations, only 76% of study participants reported wearing a mask more often during the pandemic. A poll conducted around the same time as this study found that 75% of the general public somewhat closely or very closely followed government guidelines to wear masks outside their homes [9]. Subsequently, two polls in the second and third weeks of May 2020 reported that 69% of Americans said they wear a mask or face covering all/most of the time or always/most of the time [5,10]. A recent poll from the first week in June 2020 found that only 65% of participants reported wearing a mask all or most of the time [11]. While the majority of Americans continue to wear masks in public, the drop in numbers is of concern. A gradual drop in people wearing masks, along with the reopening of business and public places and the growing travel and outings warrant attention from public health practitioners and policymakers. Especially, when in October 2020, the majority of the American states reported a sudden uptick in cases. The sustained and repeated emphasis on preventive measures is much needed despite the “mask fatigue” and “quarantine fatigue” that is being reported across the U.S. [12,13].

Significant differences were found in this study for wearing masks and gloves in the early stages of the pandemic by sex and age with females and older groups more likely to report these practices. This finding is confirmed by polls and media reports during the same time as this study across the U.S. In these polls, women—both Democrats and Republicans—are more likely to wear masks in public compared to men of the same political affiliation [5,9,10,11]. According to a recent study, men have higher negative emotions (e.g., shame, guilt, and stigma) associated with wearing masks and are less likely to believe that COVID-19 poses a serious threat [14]. Similarly, in polls nationwide, younger individuals (≤36 years of age) are less likely to report wearing masks and gloves and perceive fewer benefits of such protective practices [2,9,10,11,14]. This is a peculiar behavioral pattern, as even before the pandemic, males and younger individuals in the U.S. were more likely to engage in riskier behaviors such as drug use and violence and less likely to practice general hygienic behaviors such as handwashing [15,16,17]. Unfortunately, according to preliminary reports, men have a higher risk of mortality from COVID-19, and deaths of younger individuals are not uncommon [17,18]. Targeted interventions to promote mask-wearing are therefore much needed for this population.

Besides sex and age, the wearing of masks was more prevalent during the pandemic (MT) in participants who lived in the Northeastern U.S., were unemployed, living with family, or were non-Hispanics and racial minorities. These findings warrant additional exploration in light of multiple sociodemographic and cultural risk factors [17,18,19,20]. For example, it could be possible that those living in the Northeastern U.S. were more likely to wear a mask due to the severity of the disease in the eastern part of the country. Racial minorities (e.g., African-Americans) and those with a family may have had to work (e.g., low paying or essential services) and go out in the public. Moreover, for certain disadvantaged groups, reports of higher COVID-19-related morbidity and mortality, the general lack of ability to pay for healthcare, and fear of catastrophic consequences may have prompted a greater prevalence of mask-wearing [17,18,19,20].

Since data for this study were collected, a few major developments have occurred [21,22,23,24]. First, the routine use of gloves has not been recommended, nor have any benefits been reported. The CDC suggests using gloves only during household cleaning and disinfection or while taking care of someone who is sick. Following these guidelines, recent studies have also found that individuals around the world are reporting the wearing of gloves in high-risk situations. Second, many states in the U.S. mandated wearing masks after our data were collected, and this may influence mask-wearing behaviors. Polls have also found more Americans now backing the requirements and mandates for wearing a mask in public places. Third, recent reports and polls have found the prevalence of wearing masks similar to our study or higher as the pandemic continues. Additional research is needed on mask-wearing in light of quarantine fatigue, the rising number of cases of COVID-19, attitudinal and behavioral factors, myths and rumors about masks, and types of masks being used in the general population (e.g., cloth versus surgical). Finally, recent reports have also explored the comfort of masks within the general population and healthcare workers (e.g., breathing issues or pressure ulcers) and myths about mask use (e.g., inhalation and accumulation of carbon dioxide) [21,22,23,24]. Continued surveillance of mask-wearing behaviors in the general population along with effective and sustained public health messaging is warranted for the general population.

The results of this study are subject to numerous potential limitations. The study results are restricted by all threats to the validity and reliability of survey study designs (e.g., reliance on self-reported behaviors, recall bias in participants, socially desirable responses, and the inability to establish cause-and-effect relationships). Moreover, there are many other characteristics of individuals (e.g., chronic disease burden) that could have played a role in whether or not an individual practiced protective behavior. Finally, a major threat to external validity is that the sample is limited in nature and extent (e.g., individuals who were familiar with the online survey study environment). This would mean that the results of the study cannot be generalized to several groups of individuals across the U.S. Despite these limitations, our study is one of the earliest and largest studies in the U.S, with robust measures of protective behaviors for COVID-19.

## 5. Conclusions

In this earliest and largest study from the U.S., we identified key sociodemographic differences in mask and glove-wearing behaviors to prevent COVID-19 spread and transmission. Additional research is warranted to explore evidence-based practices and effective risk communication strategies to increase the practice of such protective behaviors, with special focus on certain groups that are vulnerable and less likely to practice the wearing of masks. Longitudinal and repeated studies are needed to assess mask -wearing over time as the COVID-19 pandemic unfolds further to assess public compliance with protective behaviors. The results of such studies with assessments for additional behavioral risk factors could help public health practitioners and researchers in designing interventions for promoting protective behaviors to reduce COVID-19 related morbidity and mortality.

## Figures and Tables

**Table 1:** Demographic Characteristics of Study Participants.

Demographic Characteristics		N (%)
**Sex**	Male	403 (48)
	Female	432 (52)
**Race**	White	529 (63)
	Asian	191 (23)
	Black	57 (7)
	Other	61 (8)
**Ethnicity**	Hispanic	180 (22)
	Non-Hispanic	655 (78)
**Employment Status**	Full time	460 (55)
	Part-time	181 (22)
	Not employed	194 (23)
**Region in the US**	Northeast	151 (18)
	Midwest	139 (17)
	South	299 (36)
	West	230 (28)
**Age**	≤35 years	541 (65)
	≥36 years	291 (35)
**Marital Status**	Single	336 (40)
	Married	378 (45)
	Engaged/Cohabitating	78 (9)
	Divorced/Widowed	43 (5)
**Living with**	Alone	159 (19)
	With Family Members	596 (71)
	With Non-Family Members	80 (10)

Total Population = 835; N (%) indicate numbers and percentages for variables.

**Table 2:** Mask and Glove Wearing Differences by Sociodemographic Characteristics.

Strata	More during the Pandemic, N (%)	Same as before the Pandemic, N (%)	Less Than before the Pandemic, N (%)
**Total Population**			
Masks	638 (76)	122 (15)	58 (7)
Gloves	255 (30)	544 (65)	39 (5)
**Gender** (*M, *G)			
Male, masks	286 (73)	73 (19)	31 (8)
Female, masks	352 (82)	49 (11)	27 (6)
Male, gloves	0 (0)	405 (100)	0 (0)
Female, gloves	255 (59)	139 (32)	39 (9)
**Race** (*M)			
Asian, face masks	138 (75)	24 (13)	22 (12)
White, face masks	406 (79)	79 (15)	32 (5)
Black, face masks	44 (79)	9 (16)	3 (6)
Other races, face masks	50 (82)	10 (16)	1 (2)
Asian, gloves	47 (25)	132 (69)	12 (6)
White, gloves	169 (32)	338 (64)	22 (4)
Black, gloves	20 (35)	34 (60)	3 (5)
Other races, gloves	19 (31)	40 (66)	2 (3)
**Ethnicity** (*M)			
Hispanic, face masks	137 (75)	36 (20)	9 (5)
Non-Hispanic face masks	501 (79)	86 (13)	49 (8)
Hispanic, gloves	51 (28)	124 (68)	8 (4)
Non-Hispanic, gloves	204 (31)	420 (64)	31 (5)
**Marital Status** (*G)			
Single, masks	258 (78)	47 (14)	26 (8)
Married, masks	284 (77)	58 (16)	25 (7)
Engaged/cohabitating, masks	59 (78)	12 (16)	5 (7)
Divorced/Widowed, masks	35 (85)	4 (10)	2 (5)
Single, gloves	92 (27)	226 (67)	18 (5))
Married, gloves	109 (29)	252 (67)	17 (4)
Engaged/cohabitating, gloves	30 (39)	46 (59)	2 (3)
Divorced/Widowed, gloves	23 (54)	18 (42)	2 (4)
**Living Arrangement** (*M)			
Alone, masks	108 (71)	28 (18)	17 (11)
With family members, masks	470 (81)	79 (14)	34 (5)
With non-family, masks	58 (73)	14 (18)	7 (9)
Alone, gloves	36 (23)	114 (72)	9 (5)
With family members, gloves	196 (33)	376 (63)	25 (4)
With non-family, gloves	23 (29)	52 (65)	5 (6)
**Employment** (*M, *G)			
Full time, masks	340 (76)	69 (15)	40 (9)
Part time, masks	134 (77)	29 (17)	12 (7)
Not working, masks	162 (85)	23 (12)	6 (3)
Full time, gloves	117 (25)	325 (71)	11 (4)
Part time, gloves	64 (36)	109 (60)	8 (4)
Not working, gloves	73 (38)	108 (56)	13 (7)
**Age** (*M, *G)			
≤35 years, masks	406 (76)	82 (16)	43 (8)
≤35 years, gloves	153 (28)	365 (67)	24 (14)
≥36 years, masks	230 (81)	40 (14)	14 (5)
≥36 years, gloves	101 (35)	177 (60)	15 (5)
**Region in the USA**			
Northeast, masks	120 (81)	23 (15)	6 (4)
Midwest, masks	108 (79)	17 (13)	9 (8)
South, masks	223 (76)	47 (16)	23 (8)
West, masks	179 (80)	30 (13)	15 (7)
Northeast, gloves	55 (36)	91 (60)	5 (3)
Midwest, gloves	43 (31)	86 (62)	10 (7)
South, gloves	93 (31)	192 (64)	15 (5)
West, gloves	63 (27)	161 (64)	8 (3)

*M indicates = *p* < 0.05 for face masks and *G indicates = *p* < 0.05 for gloves.
